# The Role of Narrative Medicine and Lean Management in Umbilical Cord Blood Donation: A Story of Success

**DOI:** 10.3390/healthcare13202567

**Published:** 2025-10-12

**Authors:** Davide Dealberti, David Bosoni, Valentina Ghirotto, Carla Pisani, Jeremy Oscar Smith Pezua Sanjinez, Barbara Fadda, Erica Roberti, Michela Testa, Guglielmo Stabile, Maria Teresa Dacquino

**Affiliations:** 1 Department of Obstetrics and Gynecology, Azienda Ospedaliero-Universitaria SS. Antonio e Biagio e Cesare Arrigo, 15121 Alessandria, Italy; ddealberti@ospedale.al.it (D.D.); dd.bosoni@gmail.com (D.B.); valentinaghirotto@ospedale.al.it (V.G.); cpisani@ospedale.al.it (C.P.); jeremyoscarsmith.pez@ospedale.al.it (J.O.S.P.S.); bfadda@ospedale.al.it (B.F.); eroberti@ospedale.al.it (E.R.); mtesta@ospedale.al.it (M.T.); mdacquino@ospedale.al.it (M.T.D.); 2 Department of Medical and Surgical Sciences, Institute of Obstetrics and Gynaecology, University of Foggia, 71122 Foggia, Italy

**Keywords:** narrative medicine, lean management, umbilical cord blood donation, patient engagement, healthcare process improvement

## Abstract

**Background/Objectives**: Umbilical cord blood (UCB) is a valuable source of hematopoietic stem cells used in treating blood and immune disorders. Despite its potential and the availability of public banking systems in Italy, donation rates remain low due to patient misinformation, emotional barriers, and organizational inefficiencies. This study aimed to evaluate the impact of integrating Narrative Medicine (NM) and Lean Management (LM) on UCB donation rates and operational effectiveness at the University Hospital of Alessandria. **Methods**: This prospective, single-center pre-post study ran from July 2022 to December 2024. Two interventions were introduced: NM training for healthcare staff to enhance empathetic communication, and LM-based reorganization of workflows to improve process efficiency. Outcomes included changes in UCB donation and adherence rates, transplant-eligible unit percentages, and patient satisfaction, assessed through institutional and project-specific surveys (PERLA–SIMeN). **Results**: Post-intervention, donation rates increased from 0% in early 2022 to 30.8% (2022), 25.8% (2023), and 30.6% (2024), with adherence rates near 40%, far exceeding the national average of ~3%. Patient satisfaction improved, resulting in PERLA certification in February 2025. **Conclusions**: The integration of NM and LM significantly improved both patient engagement and organizational efficiency. Empathetic communication fostered trust and reduced emotional barriers, while LM optimized workflows and resource use. These results suggest the model is applicable in other hospitals to enhance UCB donation outcomes and overall quality of maternal care.

## 1. Introduction

Umbilical cord blood (UCB) donation represents a critical resource in modern medicine due to its richness in hematopoietic stem cells, which serve as progenitors for all blood cell lineages [1,2]. These cells provide established, life-saving treatments for various severe congenital and acquired hematological disorders, such as leukemia, as well as immune deficiencies [1,2]. Beyond hematology recent research has expanded the therapeutic potential of UCB to include regenerative treatments for treating high-prevalence and high-mortality conditions including cardiovascular diseases [3], neurodegenerative disorders [4], and diabetes [5].

Umbilical cord blood (UCB) donation can be for allogenic solidaristic purposes, and in this case the Blood Units are stored in specific public Blood Banks, or for private purposes (autologous or family use) [6]. While the value of storing UCB in public banks for transplantation or research purposes is nowadays clear, the debate on private collection is still open and wide-ranging [7].

Despite the advancing knowledge beneath this practice, the advances in scientific research, and the regulations implemented in many countries, increasing donation rates through greater involvement of expectant mothers and healthcare professionals remains a complex challenge even today [8,9].

In Italy, the Italian Cord Blood Network (ITCBN), established in 2009, coordinates 18 public banks across 13 regions, connected to 269 collection centers. Although these centers account for 64% of national births, voluntary donation rates remain low, averaging below 3%, and only approximately 5% of collected units meet transplantation criteria and can be banked [10,11]. Barriers include informational gaps, concerns for newborn safety, social factors, and logistical difficulties, often exacerbated by the organizational model where external midwives recruit donors sporadically, limiting effective patient engagement.

In our country, UCB recruitment is frequently managed by external midwives funded through temporary research grants, who operate outside regular obstetric care workflows. This structural limitation reduces opportunities for trust-building and patient interaction, thus hindering effective donor recruitment. Within this context, two complementary approaches have gained attention for addressing the human and operational challenges in healthcare delivery: Narrative Medicine (NM) and Lean Management (LM). NM fosters empathetic listening and the co-construction of care pathways through patient storytelling, thereby improving communication and trust between patients and healthcare providers [12]. LM, originally developed for industrial efficiency, is increasingly applied in healthcare to identify and reduce inefficiencies, optimize organizational processes, may enhance donation rates by improving both patient engagement and operational efficiency [13]. For these reasons in 2022 in our center it was decided to apply these 2 techniques to improve recruitment results.

This study investigates the combined application of NM and LM within the Obstetrics and Gynecology Department of the University Hospital of Alessandria, assessing its impact on UCB donation rates, patient satisfaction, and organizational performance. Our findings suggest that integrating these approaches creates a potentially applicable model to enhance both patient-centered care and operational efficiency in maternal health services.

## 2. Materials and Methods

### 2.1. Study Design and Setting

This prospective, single-center pre-post study was conducted at the Department of Obstetrics and Gynecology of the University Hospital of Alessandria, Italy. This research was approved by the Institutional Review Board of the University Hospital of Alessandria (n° 0021513). The study spanned from 1 July 2022 to 31 December 2024. The primary objective was to assess whether the integration of Narrative Medicine (NM) and Lean Management (LM) approaches would improve umbilical cord blood (UCB) donation rates, as well as increase patient satisfaction and the perceived quality of care.

The secondary objective was to evaluate the quality of collected UCB units, defined as the proportion of donated units eligible for hematopoietic transplantation, according to standards established by the national cord blood banking guidelines.

### 2.2. Participants (Study Population)

A total of 2636 women who gave birth during the study period were included. Eligibility criteria for UCB donation followed national protocols and excluded individuals with medical or obstetric contraindications (e.g., infections, autoimmune conditions, fever during labor, or preterm birth).

### 2.3. Intervention

The intervention involved two key components:

#### 2.3.1. Narrative Medicine Integration

The NM integration initiatives built upon our earlier experiences during the COVID-19 pandemic, which had revealed the importance of narrative-based care in supporting isolated mothers [14].

**Figure 1:**
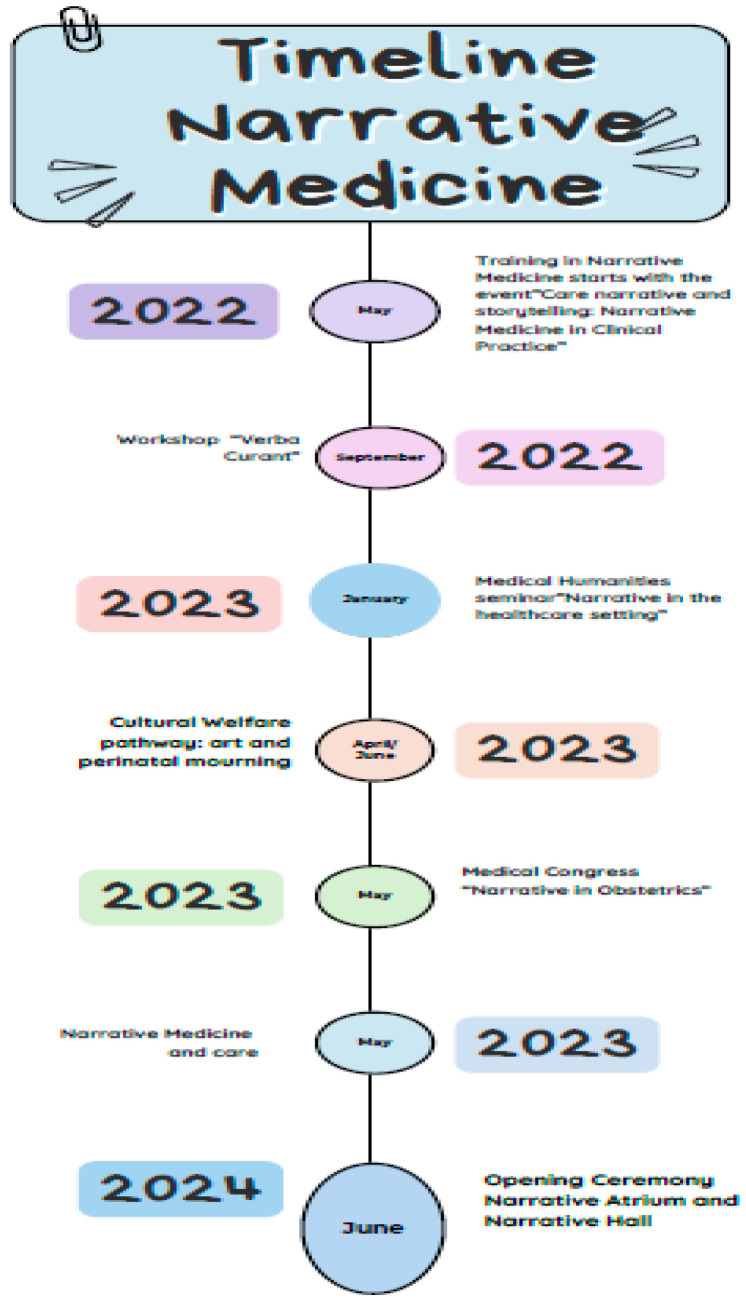
Phases of Narrative Medicine Training and Educational Methodologies.

Healthcare professionals—including physicians, midwives, and nurses, all employees of University Hospital of Alessandria—underwent NM training in collaboration with the Italian Society of Narrative Medicine (SIMeN). The training aimed to enhance empathetic listening, trust-building, and patient engagement through structured narrative workshops. These included close reading and reflection exercises using literature, visual arts, and cinema (Figure 1). All the healthcare professionals involved in the process, upon interview, declared that the NM implementation process was perceived as positive and useful. The major challenge was represented by the time required for the trainings: to this effect Staff Coordinators help was crucial.

**Figure 2:**
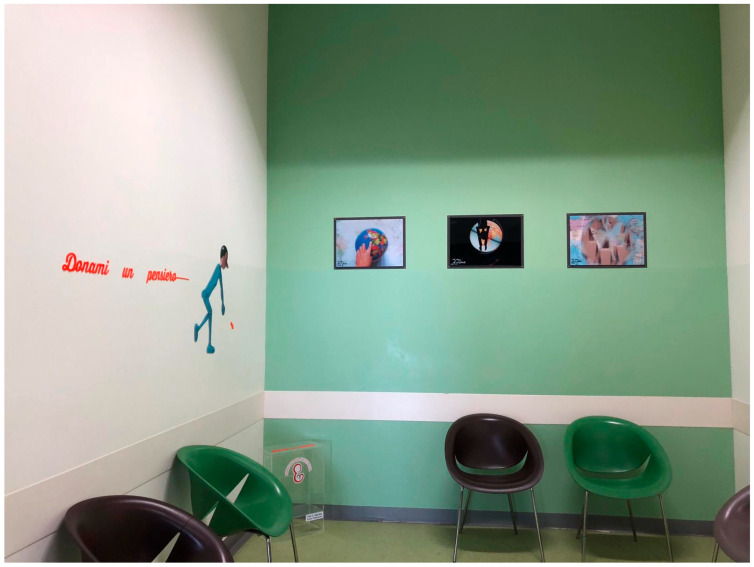
The Narrative Atrium: A storytelling-based waiting area for patients and families.

**Figure 3:**
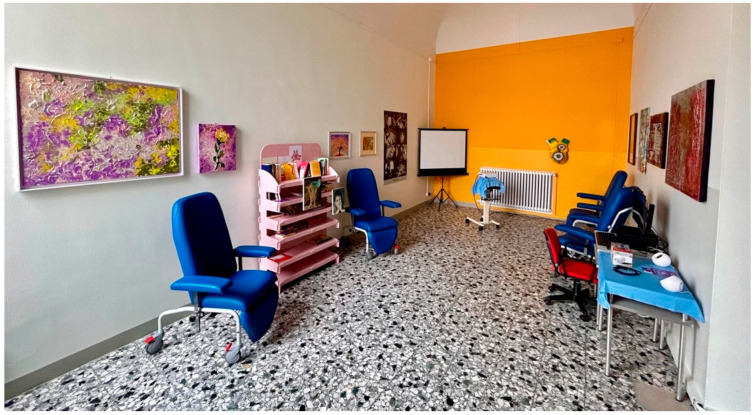
Narrative Hall. Legend: The Narrative Hall: Resources and tools for therapeutic storytelling and reflection.

The hospital established two dedicated physical spaces to foster narrative practice:

- **Narrative Atrium** (Figure 2): A redesigned waiting area encouraging storytelling and relational engagement through an interactive journey.
- **Narrative Hall** (Figure 3): A therapeutic space for emotional expression through books, films, artwork, and narrative journals.

#### 2.3.2. Lean Management Implementation

In 2019, the department obtained “Advanced Level” certification in Lean Healthcare Management. Lean principles were applied to reorganize UCB donation workflows, emphasizing process mapping, waste reduction, and value creation for patients [15,16]. This included transitioning from reliance on an externally funded midwife via ADISCO (Italian Association of Female Cord Blood Donors) to a multidisciplinary, in-house care team.

Key Lean components included:

- Staff training and engagement
- Process flow mapping and optimization
- Standardization of procedures
- Continuous quality improvement
- Enhanced communication strategies

Informational and consent materials were incorporated into the hospital’s Birth Pathway and disseminated at multiple prenatal care points, including the first pregnancy visit, the ultrasound outpatient offices, the high-risk pregnancy outpatient clinic, the 34-week Health Check-Up Clinic, and during birth preparation classes hosted in our department. In order to guarantee LM implementation at all levels, a Small Working Group including Staff (Midwifes, Nurses) Coordinators, Physician’s Director and one representative for each category was created, and managed the transition perfectly. ADISCO was involved in the process, and the information’s sharing was considered positive from the Association point of view.

### 2.4. Data Collection

Data were collected from institutional clinical records, donation registries, and patient-reported feedback. The following indicators were monitored:

- **Number of Donations**: Number of successful donations.
- **Donation Rate**: Number of women who consented to donate UCB out of total births.
- **Patient Satisfaction and Perceived Quality of Care**: Measured via corporate satisfaction questionnaires and “PERLA–Person-Centered Care” surveys (https://www.certificazioneperla.it, accessed on 20 May 2025) in collaboration with SIMeN (Italian Society of Narrative Medicine).
- **Transplant Eligibility**: Proportion of collected UCB units banked for clinical use.
- **Adherence Rate**: Percentage of pregnant women who consented to the UCB donation during our Hospital’s Birth Pathway among the women who gave birth in our Hospital in the period July 2022–2024

### 2.5. Ethical Considerations

The study followed the principles of the Declaration of Helsinki. All participants provided informed consent for donation and participation in satisfaction assessments. Ethical approval was obtained from the institutional ethics committee of the University Hospital of Alessandria.

### 2.6. Data Analysis

Descriptive statistical analysis was performed to compare pre- and post-intervention outcomes. Donation rates, satisfaction scores, and transplant eligibility percentages were examined to evaluate the intervention’s effectiveness in enhancing patient-centered care and operational performance.

## 3. Results

### 3.1. Participant Characteristics

Between 1 July 2022 and 31 December 2024, a total of **2636 women** gave birth at the University Hospital of Alessandria. All participants received information regarding umbilical cord blood (UCB) donation as part of their care pathway. No significant changes in the overall number of births or patient demographic profile were observed compared to previous years.

### 3.2. Improvement in Donation Rates

Following the implementation of the Narrative Medicine and Lean Management interventions, there was a notable increase in UCB donation rates.

Specifically, as shown in Table 1 and Table 2:

- Pre-intervention donation rate (2007–June 2022, *mean*): **3.93%**
- Post-intervention donation rate (July 2022–December 2022): **30.8%**
- Post-intervention donation rate (2023): **25.8%**
- Post-intervention donation rate (2024): **30.6%**
- Donation rate during study period (July 2022–2024, *mean*): **29.07%**

This represents a **noteworthy relative increase** in the mean donation rate compared to the previous years, suggesting improved patient engagement and organizational efficiency. It is important to underline that the donation rate in 2021 and in the first six months of 2022 was 0, this due to the COVID-19 pandemics.

In addition, we also registered the percentage of pregnant women who consented to the UCB donation during our Hospital’s Birth Pathway among the 2636 women who gave birth in our Hospital: **40.4%** between July and December 2022, **39.8%** in 2023 and **41.6%** in 2024. Of course, not all the women who agreed with donation could effectively do it, in most cases because of obstetrical contraindications occurred during the term of the pregnancy or the labour (e.g., infections, fever during labour, or preterm birth), and moreover we do not have data about the percentage of pregnant women who consented to the UCB donation between 2007 and June 2022. However, these numbers also suggest that the implementation of NM and LM in our clinical practice is effective in the enrolment process.

### 3.3. Collection and Transplant Eligibility

Of the 758 UCB donations collected post-intervention:

- **Units eligible for transplant**: 29 units (3.75% of donations, *mean*) met the national quality criteria for transplantation, as assessed by the Regional Cord Blood Bank in Turin (Table 3). This mean eligibility rate is just below the 2022 and 2023 national average (5–5.3% respectively) [10,11].

The eligibility criteria for the Regional Cord Blood Bank in Turin are:

- The medical history of the father and mother of the newborn must be known, as well as that of their respective families
- No risk of transmission of genetic diseases
- No positivity and/or risk of HIV and/or hepatitis
- No previous travel to countries with risk of endemic diseases
- No use of contraindicated medications, alcohol or drug abuse
- Gestation that must have exceeded 37 weeks
- Give birth in an accredited birthing center
- Birth without fetal distress

### 3.4. Patient Satisfaction and Perceived Quality of Care

Post-intervention evaluation using both institutional satisfaction surveys and the “PERLA–Person-Centered Care” questionnaires indicated:

- **90.3%** of patients reported feeling well-informed about UCB donation
- **88.7%** indicated they felt “actively listened to” by the midwifery team
- **92.5%** rated their overall care experience as “excellent” or “very good”

Qualitative feedback highlighted the importance of the Narrative Atrium and Hall in promoting emotional safety and enhancing trust during the birth experience. Corporate customer satisfaction surveys revealed progressive improvement in patient-reported outcomes over the two-year intervention period. In particular, ratings related to the quality of the operator-patient relationship, clarity of communication, and emotional support (these three items were the core domains of the PERLA questionnaire) showed statistically significant gains.

The department also participated in the national PERLA–SIMeN (Personalized and Person-Centered Care) initiative. Patient feedback collected through PERLA-specific tools led to the **official PERLA Certification** being awarded to the Department of Obstetrics and Gynecology on **28 February 2025**, recognizing its excellence in patient-centered care practices.

### 3.5. Operational and Organizational Improvements

The integration of Lean Management principles led to several improvements in workflow and care delivery:

- **Reduction in missed donation opportunities** due to more consistent patient engagement throughout the prenatal pathway
- **Increased internal staff autonomy**: trained in-house midwives replaced reliance on an external figure, ensuring continuity and sustainability of care **and elimination of annual external grant costs** (€5000) for a dedicated midwife (even if it is important to underline that we have not estimated the cost of the internal workforce)
- **Shortened collection procedure times**, due to smoother and standardized processes
- **Increased process reliability and safety**, as reported in internal observations. Staff reported greater motivation and ownership of the donation process, while delays caused by prior inefficiencies were notably reduced.
- **Standardized communication protocols** and training improved interdepartmental coordination and reduced delays in consent and collection logistics

These organizational changes contributed to a more robust and patient-centered UCB donation process.

### 3.6. Perception of Emotional and Informational Support

The empathetic communication approach introduced through Narrative Medicine training was positively received by patients. Survey data and narrative feedback qualitatively indicated:

- Increased **trust in healthcare professionals**
- Greater **participation in decision-making**
- Enhanced **perception of being welcomed and understood**

## 4. Discussion

This study demonstrates the effectiveness of an integrated approach combining Narrative Medicine (NM) and Lean Management (LM) in improving outcomes related to umbilical cord blood (UCB) donation within a hospital-based obstetric setting. Following the implementation at the University Hospital of Alessandria, we observed a significant increase in donation rates, improved patient satisfaction, enhanced quality of collected units, and greater organizational efficiency.

### 4.1. Integration of Humanistic and Operational Models

The use of **Narrative Medicine** contributed to a more empathetic and trust-based clinician–patient relationship, which literature suggests is a key factor in increasing voluntary health behaviors [12,17]. Training programs and the establishment of narrative spaces (e.g., the Narrative Atrium and Hall) created environments where patients felt heard, supported, and better informed, thus reducing emotional barriers to participation [18].

Simultaneously, the application of **Lean Management** principles helped optimize workflow efficiency and staff engagement, aligning with previous research showing that LM can reduce waste and enhance care quality in obstetric and gynecological services [15,16,19]. By eliminating the dependency on externally funded personnel and integrating recruitment into routine care, the model became more sustainable and applicable.

### 4.2. Comparison with National and International Data

The post-intervention **mean donation rate of 29.7%** compare favorably with national Italian averages (2.98%) [10,11]. This outcome support the hypothesis that informational and organizational barriers, rather than clinical limitations, are the primary factors inhibiting public UCB donation—an argument also supported by earlier findings in Italy and other countries [6,7,8]. The post-intervention mean UCB eligibility rate of 3.75% is just below the 2022 and 2023 Italian averages (5–5.3% respectively). This outcome needs further analysis (that is ongoing) and suggests to improve the collection practical method in our Hospital.

### 4.3. Implications for Healthcare Delivery

This model highlights the potential of interdisciplinary strategies to improve both **clinical outcomes** and **patient experience**. The convergence of NM and LM addresses two traditionally separate domains—empathy and efficiency—proving that they can be mutually reinforcing when implemented cohesively. Moreover, the approach aligns with the **person-centered care framework** endorsed by PERLA–SIMeN, suggesting broader applicability across maternal health services.

### 4.4. Limitations

This study was conducted in a single center and may reflect context-specific strengths such as strong leadership, existing NM culture, and prior Lean certification. Additionally, the absence of a randomized control group limits causal inference. Nonetheless, the pre-post design allowed for real-time and real-life implementation and adjustment.

### 4.5. Future Directions

Further multi-center studies are warranted to validate the generalizability of these findings and assess long-term sustainability. Future research should also investigate the cost-effectiveness of this integrated model and its applicability in other areas of maternal–child healthcare, such as breastfeeding support or perinatal mental health.

## 5. Conclusions

The integration of **Narrative Medicine** and **Lean Management** within the Department of Obstetrics and Gynecology at the University Hospital of Alessandria led to measurable improvements in **umbilical cord blood donation rates**, **patient satisfaction**, and **organizational efficiency**. By combining empathetic patient engagement with streamlined operational workflows, the project successfully addressed both human and systemic barriers to UCB collection.

These findings offer a promising model for enhancing **patient-centered care** and **clinical outcomes** in maternal health settings. The success of this intervention suggests that multidimensional approaches—rooted in both communication and process management—can significantly improve participation in public health initiatives like UCB donation. Broader adoption of similar models, supported by institutional commitment and interprofessional collaboration, could help bridge the gap between scientific opportunity and real-world implementation in obstetrics.

## Figures and Tables

**Table 1:** Umbilical Cord Blood Donation Rates at the University Hospital of Alessandria from 2007 to June 2022.

Year	Number of Births	Number of Donations	Donation Rate
2007	1222	123	10.06%
2008	1356	102	7.50%
2009	1349	41	3.03%
2010	1298	113	8.70%
2011	1301	112	8.21%
2012	1364	65	4.76%
2013	1332	46	3.45%
2014	1423	18	1.26%
2015	1395	16	1.14%
2016	1307	9	0.68%
2017	1311	15	1.14%
2018	1180	20	1.69%
2019	1213	54	4.38%
2020	1085	26	2.39%
2021	1031	0	0%
1 January–30 June 2022	516	0	0%

**Table 2:** Umbilical Cord Blood Donation Rates at the University Hospital of Alessandria from July 2022 to 2024.

Year	Number of Births	Number of Donations	Donation Rate
1 July–31 December 2022	584	180	30.8%
2023	1046	270	25.8%
2024	1006	308	30.6%

**Table 3:** Percentage of Cord Blood Units Eligible for Transplantation (2022–2024).

Year	Number of Units Donated	Eligible Units	Eligible Units Rate
1 July 2022–31 December 2022	180	5	2.77%
2023	270	15	5.55%
2024	308	9	2.92%

## Data Availability

The authors confirm that the data supporting the findings of this study are available within the article.

## Abbreviations

The following abbreviations are used in this manuscript:

UCB	Umbilical cord blood
NM	Narrative Medicine
LM	Lean Management
SIMeN	Italian Society of Narrative Medicine
ADISCO	Italian Association of Female Cord Blood Donors
